# Non-Invasive Ventilation for Community-Acquired Pneumonia: Outcomes and Predictors of Failure from an ICU Cohort

**DOI:** 10.3390/medicina60010081

**Published:** 2023-12-30

**Authors:** Adam Watson, Sina Yadollahi, Alexander Fahmy, Sania Mahar, Dominic Fritche, Ryan Beecham, Kordo Saeed, Ahilanandan Dushianthan

**Affiliations:** 1 General Intensive Care Unit, University Hospital Southampton NHS Foundation Trust, Southampton SO16 6YD, UK; a.watson@soton.ac.uk (A.W.); sania.mahar@uhs.nhs.uk (S.M.); ryan.beecham@uhs.nhs.uk (R.B.); 2 Clinical and Experimental Sciences, Faculty of Medicine, University of Southampton, Southampton SO17 1BJ, UK; dominic.fritche@uhs.nhs.uk (D.F.); kordo.saeed@uhs.nhs.uk (K.S.); 3 Department of Microbiology, University Hospital Southampton NHS Foundation Trust, Southampton SO16 6YD, UK; 4 Perioperative and Critical Care Theme, NIHR Southampton Biomedical Research Centre, University Hospital Southampton NHS Foundation Trust, Southampton SO16 6YD, UK

**Keywords:** pneumonia, respiratory failure, non-invasive ventilation, NIV, HACOR, SOFA

## Abstract

*Background and Objectives*: The use of non-invasive ventilation (NIV) for community-acquired pneumonia (CAP) remains controversial. NIV failure in the setting of acute hypoxemic respiratory failure is associated with increased mortality, highlighting the need for careful patient selection. *Methods and Methods*: This is a retrospective observational cohort study. We included 140 patients with severe CAP, treated with either NIV or invasive mechanical ventilation (IMV) as their primary oxygenation strategy. *Results:* The median PaO_2_/FiO_2_ ratio and SOFA score upon ICU admission were 151 mmHg and 6, respectively. We managed 76% of patients with NIV initially and report an NIV success rate of 59%. Overall, the 28-day mortality was 25%, whilst for patients with NIV success, the mortality was significantly lower at 13%. In the univariate analysis, NIV failure was associated with the SOFA score (OR 1.33), the HACOR score (OR 1.14) and the presence of septic shock (OR 3.99). The SOFA score has an AUC of 0.75 for NIV failure upon ICU admission, whilst HACOR has an AUC of 0.76 after 2 h of NIV. *Conclusions*: Our results suggest that a SOFA ≤ 4 and an HACOR ≤ 5 are reasonable thresholds to identify patients with severe CAP likely to benefit from NIV.

## 1. Introduction

### 1.1. Background

Community-acquired pneumonia (CAP) is a leading cause of hospitalization and mortality globally [1]. The serious complications of CAP include sepsis and acute respiratory failure (ARF), both of which may require intensive care unit (ICU) admission. Over the past decade, the use of non-invasive ventilation (NIV) as a respiratory support strategy for CAP with associated primary hypoxemic respiratory failure has increased, [2,3,4] despite a lack of strong evidence on its efficacy. Although NIV is accepted as the first-line respiratory support for patients with hypercapnic respiratory failure or acute heart failure, its use for CAP remains controversial, and guidelines generally do not support its routine use [5,6]. However, more recently, the use of helmet continuous positive airway pressure (CPAP) and NIV has been shown to be effective in patients with hypoxemic respiratory failure in improving outcomes [7,8].

In patients with pneumonia, NIV appears to improve oxygenation and, in those who respond, may reduce the requirement for invasive mechanical ventilation (IMV) and mortality [9,10]. However, the success of NIV in the context of ARF associated with severe CAP ranges from 20 to 76%, and selecting patients who will respond to NIV is a challenge [11]. The failure of NIV appears to be associated with the type of pneumonia, the disease severity, physiological derangement, the presence of other organ failures and worsening oxygenation [12,13,14,15,16,17,18,19]. Furthermore, NIV failure and the delayed initiation of IMV may increase mortality and complications associated with intubation [18,20,21]. The need for careful patient selection and early identification of those at risk of NIV failure is therefore clear. In response to these findings, the Heart Rate, Acidosis, Consciousness, Oxygenation and Respiratory Rate (HACOR) score was developed for patients with hypoxemic ARF [22]. Although the HACOR score appears to predict NIV failure in unselected hypoxemic respiratory failure [23,24] and more recently in COVID-19 [25,26,27], its predictiveness for NIV failure has yet to be studied exclusively in patients with CAP.

### 1.2. Aims and Objectives

We aimed to investigate the outcomes of NIV use and predictors of NIV failure in patients with ARF secondary to severe CAP. Our primary objective was to report the outcomes of NIV use in this context. Our secondary objectives were to identify whether the HACOR score, Sequential Organ Failure Assessment (SOFA) score, or Ratio of Oxygen Saturation index (ROX index) predict NIV failure for severe CAP cases in an ICU setting.

## 2. Materials and Methods

### 2.1. Study Design and Setting

In this single-center retrospective cohort study, we included consecutive adults with CAP admitted to our ICU prior to the COVID-19 pandemic. University Hospital Southampton is a large tertiary hospital in the south of England serving 1.9 million people. The study data were collected for the period between 1st February 2016 and 30th April 2017. University Hospital Southampton NHS Foundation Trust (RHM CRI 0370) sponsored this study, and ethical approval was obtained from the NHS Health Research Authority (IRAS 232922). The study is compliant with local ethical standards, and no identifiable patient data are presented here. This manuscript complies with STROBE guidelines [28].

### 2.2. Inclusion and Exclusion Criteria

We identified eligible patients by searching our electronic patient records (MetaVision CIS, iMDsoft, Tel Aviv, Israel) by diagnosis upon ICU admission. Our inclusion criteria were (1) a diagnosis of CAP, (2) ARF requiring respiratory support on an ICU and (3) a PaO_2_/FiO_2_ (P/F) ratio ≤ 300 mmHg. We excluded patients who received high-flow nasal oxygen (HFNO) as their sole first respiratory support; however, we included patients who received intermittent HFNO to facilitate breaks during NIV. 

### 2.3. Data Collection

Anonymized patient data were retrieved from our electronic patient records. The data collected included demographic information, co-morbidities (described using Charlson’s Comorbidity Index, CCI) [29] and laboratory values upon ICU admission. We categorized co-morbidities as ischemic heart disease (IHD), congestive cardiac failure (CCF), chronic obstructive pulmonary disease (COPD), other chronic respiratory diseases, cerebrovascular disease, diabetes mellitus, chronic kidney disease (3b or worse), active cancer (solid organ or hematological) or immunocompromise (active cancer or immunosuppressive medication). Acute Physiology and Chronic Health Evaluation 2 (APACHE II) and SOFA scores were calculated at various timepoints [30,31]. Our exposure variable was the type of respiratory support used upon ICU admission (NIV vs. IMV), which was entirely dependent on clinician choice. NIV included both continuous positive airway pressure (CPAP) and bilevel positive airway pressure (BIPAP). We also categorized patients who received NIV first as either NIV success (defined as discharged alive from the ICU) or NIV failure (defined as a requirement for IMV or death). For patients who received NIV first, we calculated the original HACOR score [22], the updated HACOR score (uHACOR) [24] and the ROX index [32] upon ICU admission and after 2 h of NIV. We defined sepsis and septic shock according to the Sepsis-3 consensus definitions [33]. The primary outcome reported is 28-day mortality from ICU admission. Our secondary outcome measures are the rate of NIV failure, the requirement for organ support, ICU mortality, ICU days and hospital days.

### 2.4. Statistical Analysis

Our data are reported using conventional descriptive statistics, with categorical data presented as numbers (percentage). We used the Kolmogorov–Smirnov test to assess continuous data for normality, and as our dataset was generally non-normally distributed, we presented continuous variables as medians (inter-quartile range; IQR). Comparisons were made between survivors and non-survivors at 28 days, between patients who received NIV and IMV as their first respiratory support and between NIV success and failure. The Mann–Whitney U test was used to compare continuous variables, and Fisher’s exact test was used for proportions between groups. We used a univariate logistic regression to investigate the relationships between variables on the dichotomous outcomes of NIV failure and 28-day mortality. Kaplan–Meier survival curves are also used to describe 28-day mortality. The ability of variables to predict NIV failure and 28-day mortality was investigated using a receiver operating characteristic (ROC) curve analysis. We used SPSS v28 (IBM Corp., Armonk, NY, USA) for our analysis. A *p*-value of <0.05 was taken to be statistically significant.

## 3. Results

### 3.1. Patient Characteristics

**Figure 1:**
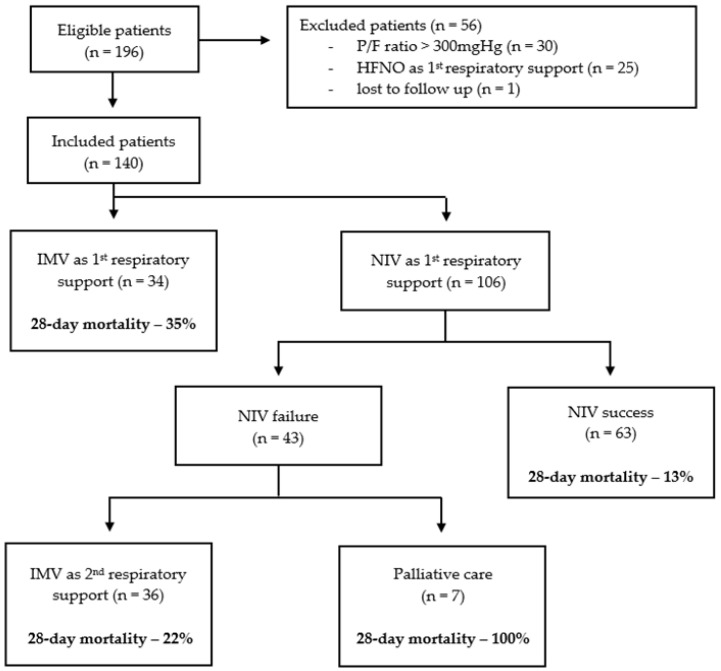
Flow diagram of eligible, included and excluded patients by respiratory support received and corresponding 28-day mortality.

Of the 196 eligible patients with CAP admitted to our ICU, we included 140 patients in our analysis (Figure 1). We excluded 25 patients as they received HFNO as their sole first respiratory support, and 30 patients were excluded as their P/F ratio upon ICU admission was >300 mmHg. A single further patient was lost to follow-up due to hospital transfer and was excluded. The median age and CCI were 65 years (IQR 54–73) and four (IQR 2–5). Overall, patients most frequently reported COPD (n = 33, 24%), other respiratory diseases (n = 31, 22%) and diabetes mellitus (n = 33, 24%) as co-morbidities (Table 1). The median APACHE II score, SOFA score and PaO_2_/FiO_2_ ratio upon ICU admission were 19 (IQR 15–23), 6 (IQR 3–10) and 151 mmHg (IQR 111–203), respectively. Upon ICU admission, 27 patients (19%) had septic shock, whilst 19 (14%) and 14 (10%) patients had evidence of ARDS or CPE on chest radiograph, respectively. The median time from hospital to ICU admission was 0 days (IQR 0–1), with 46% (n = 64) of patients admitted from the Emergency Department and 52% (n = 73) from hospital wards.

The overall 28-day mortality was 25% (n = 35). We noted that survivors were younger (64 vs. 69 years, *p* = 0.002), less comorbid (CCI 3 vs. 4, *p* = 0.002) and had lower white cell counts (12.4 vs. 17.9 × 10^9^/L, *p* = 0.033; WCC) and APACHE II scores (18 vs. 23, *p* = 0.001) upon ICU admission. There were no other differences in patient demographics or laboratory values, although the SOFA score appeared to tend to be lower in survivors (Table 1). In the univariate analysis, the 28-day mortality was associated with age (OR 1.05, 95% CI 1.02–1.09, *p* = 0.002), CCI (OR 1.41, 95% CI 1.13–1.75, *p* = 0.002) and APACHE II score (OR 1.14, 95% CI 1.05–1.23, *p* = 0.002), but not with NIV use (OR 0.51, 95% CI 0.22–1.18, *p* = 0.115). 

### 3.2. Type of Respiratory Support

There were 34 patients (24%) who received IMV and 106 patients (76%) who received NIV as their first respiratory support (Table 2). Overall, the patients who received IMV first were older (71 vs. 63, *p* = 0.023), reported fewer non-COPD respiratory comorbidities (9% vs. 26%, *p* < 0.034) and had a higher prevalence of de novo ARF (76% vs. 53%, *p* = 0.017). We also noted that those who received IMV had higher SOFA scores (11 vs. 5, *p* < 0.001) and a greater prevalence of septic shock upon ICU admission (32% vs. 15%, *p* = 0.043). The univariate analysis showed that the decision to start IMV was associated with the presence of non-COPD respiratory comorbidities (OR 0.27, 95% CI 0.08–0.95, *p* = 0.42), de novo ARF (OR 2.90, 95% CI 1.20–6.99, *p* = * 0.018), septic shock (OR 2.69, 95% CI 1.1–6.58, *p* = * 0.030) and SOFA score (OR 1.57, 95% CI 1.34–1.83, *p* < * 0.001).

**Figure 2:**
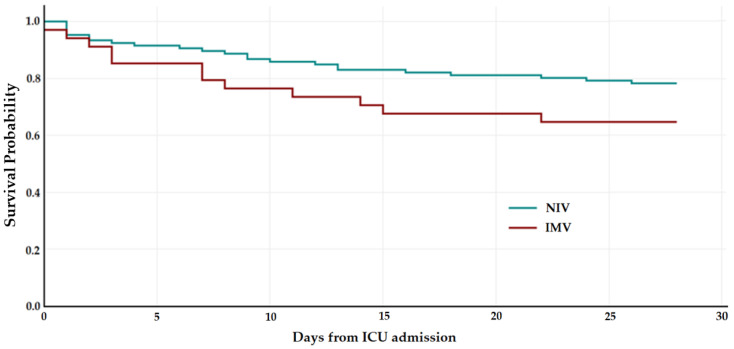
Kaplan–Meier survival curve according to type of first respiratory support. Abbreviations: non-invasive ventilation (NIV), invasive mechanical ventilation (IMV).

There was a substantial difference in the 28-day mortality over time between patients who received IMV or NIV first (Figure 2). The ICU mortality was greater for IMV than NIV (29% vs. 13%, *p* = 0.038), with a trend towards a higher 28-day mortality for IMV (35% vs. 22%, *p* = 0.11). Furthermore, the requirements for cardiovascular support or renal replacement therapy (RRT) were greater for patients who received IMV (Table 2). We also noted that patients who received IMV first had a longer ICU stay (8 vs. 5 days, *p* = 0.006), although there was no difference in overall hospital stay (*p* = 0.162).

### 3.3. NIV Outcomes

We compared outcomes between patients with NIV success and failure (Table 3). Of the 106 patients who received NIV as their first respiratory support, 63 (59%) survived to ICU discharge (i.e., NIV success). The remaining 43 patients (41%) failed NIV and either required IMV as their second respiratory support (n = 36, 34%) or were not eligible for IMV and died subsequently (n = 7, 7%). In patients with NIV success, the prevalence of diabetes mellitus was greater (33% vs. 14%, *p* = 0.040), whilst the prevalence of septic shock was lower (8% vs. 26%, *p* = 0.025). However, there were no other differences in patient demographics, comorbidities or the prevalence of ARDS, CPE, or de novo ARF. 

The median time to NIV failure was 24 h (IQR 8–36 h). Overall, patients with NIV failure had a higher ICU mortality (33% vs. 0%, *p* < 0.001), longer ICU stays (8 vs. 4 days, *p* < 0.001) and a higher 28-day mortality (35% vs. 13%, *p* = 0.009). Furthermore, there was no difference in 28-day mortality between patients who received IMV first and patients who failed NIV before receiving IMV second (35% vs. 22%, *p* = 0.293). Overall, when patients with NIV success were compared to all other patients, we noted a lower 28-day mortality (13% vs. 35%, *p* = 0.003) and a shorter length of ICU stay (4 vs. 8 days, *p* < 0.001).

### 3.4. Predictors of NIV Failure

We compared laboratory tests and prognostic scores at various timepoints between NIV success and failure (Table 3). After 2 h of NIV, the HACOR score and ROX index data were available for 78% (n = 83) and 84% (n = 89) of patients, respectively. Although non-significant, the NIV failure group had a lower P/F ratio and ROX index. 

The SOFA score was higher in patients with NIV failure at both ICU admission (7 vs. 4, *p* < 0.001) and after 24 h (8 vs. 3, *p* < 0.001). The prevalence of NIV failure was 12%, 32%, 36%, 62% and 75% for SOFA scores upon ICU admission of ≤2, 3–4, 5–6, 7–8 and ≥9, respectively. Upon ICU admission, when a threshold for the SOFA score of ≤4 to predict NIV success was adopted, we found that the sensitivity, specificity, positive predictive value and negative predictive value were 61% (95% CI 47–73), 72% (95% CI 56–84), 76% (95% CI 65–84, positive likelihood ratio 2.17) and 56% (95% CI 47–65, negative likelihood ratio 0.55), respectively. In the univariate analysis, the SOFA score upon ICU admission and after 24 h had ORs for NIV failure of 1.33 (95% CI 1.15–1.54, *p* < 0.001) and 1.52 (95% CI 1.27–1.81, *p* < 0.001). Furthermore, the presence of septic shock upon ICU admission had an OR of 3.99 for NIV failure (95% CI 1.27–12.5, *p* = 0.018). 

The HACOR score was higher for NIV failure both at the time of ICU admission (6 vs. 5, *p* = 0.007) and after 2 h (10 vs. 5, *p* = 0.001). After 2 h of NIV, the prevalence of NIV failure was 11%, 38% and 55% for patients with original HACOR scores of ≤3, 4–6 and ≥7, respectively. If a threshold for the HACOR score of ≤5 after 2 h is adopted to predict NIV success, we found that the sensitivity, specificity, positive predictive value and negative predictive value were 53% (95% CI 38–67), 85% (95% CI 69–95), 84% (95% CI 69–92, positive likelihood ratio 3.61) and 56% (95% CI 48–64, negative likelihood ratio 0.55), respectively. In the univariate analysis, the HACOR score upon ICU admission and after 2 h had ORs for NIV failure of 1.14 (95% CI 1.04–1.24, *p* = 0.004) and 1.13 (95% CI 1.04–1.22, *p* = 0.004), respectively.

The updated HACOR score was also higher for NIV failure both at the time of ICU admission and after 2 h (Table 3). After 2 h of NIV, the prevalence of NIV failure was 7%, 33%, 38% and 65% for patients with uHACOR scores of ≤7, 7.5–10.5, 11–14.5 and ≥15, respectively. Therefore, if a threshold for the uHACOR of ≤ 10 after 2 h to predict NIV success is adopted, the sensitivity, specificity, positive predictive value and negative predictive value are 44% (95% CI 29–59), 88% (95% CI 73–97), 84% (95% CI 66–93, positive likelihood ratio 3.72) and 53% (95% CI 46–59, negative likelihood ratio 0.64), respectively. In the univariate analysis, the uHACOR score upon ICU admission and after 2 h had ORs for NIV failure of 1.14 (95% CI 1.06–1.23, *p* < 0.001) and 1.15 (95% CI 1.07–1.24, *p* < 0.001), respectively.

**Figure 3:**
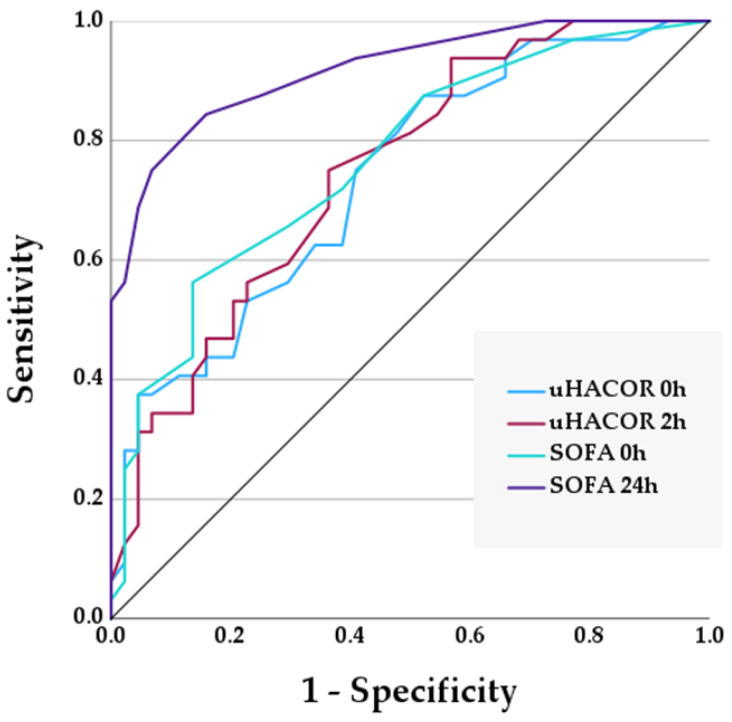
Receiver operating characteristic (ROC) curves for uHACOR and SOFA scores.

We also report the area under the ROC curve (AUC) for the ability of prognostic scores to predict NIV failure (Table 4 and Figure 3). The SOFA score predicts NIV failure upon ICU admission with an AUC of 0.749 (95% CI 0.655–0.844, *p* < 0.001) and after 24 h with an AUC of 0.819 (95% CI 0.725–0.914, *p* < 0.001). Furthermore, the uHACOR score predicts NIV failure upon ICU admission with an AUC of 0.717 (95% CI 0.617–0.818, *p* < 0.001) and after 2 h with an AUC of 0.762 (95% CI 0.660–0.863, *p* < 0.001). However, neither the original HACOR nor ROX index can better predict NIV failure at any timepoint.

## 4. Discussion

In this single-center retrospective cohort study, we report our use of NIV for patients with ARF secondary to severe CAP. Despite the lack of guidelines, the use of NIV for this purpose is now relatively common [2,3,4], and the most recent literature suggests that NIV may reduce the requirement for IMV and mortality [7,8,9,10]. However, NIV failure is associated with increased mortality [18,20], and it remains unclear which patients are most likely to benefit. We found that the SOFA and HACOR scores, but not the ROX index, can be used to accurately predict NIV failure (Table 4). These findings are broadly consistent with previous studies on prognostic scores in patients with hypoxaemic ARF [14,22,24]. Although the study was retrospective and single-centered, the findings show that NIV may benefit patients with severe CAP.

The common aetiology of ARF in this cohort was severe CAP, with a median P/F ratio upon ICU admission of 151 mmHg, suggesting moderate hypoxemic respiratory failure. Our inclusion criteria were based upon the diagnosis of CAP, as opposed to hypoxemic ARF more broadly, as we believed that aetiology-specific results would be of more relevance to clinical decision making. The median SOFA score upon ICU admission was six, which suggests that most patients had additional non-pulmonary organ dysfunction secondary to sepsis. The disease severity in this cohort is broadly comparable to other previous similar studies [12,13,18].

In our cohort, the overall 28-day mortality was 25%. We found that non-survivors were older, more comorbid and had higher WCCs and APACHE II scores upon ICU admission. In total, 76% of the patients received NIV as the first mode of respiratory support, and the rest (24%) received IMV first. Overall, those who received IMV first had higher SOFA scores and WCCs upon ICU admission, which may suggest that this decision was based upon the presence of another organ dysfunction. The prevalence of de novo ARF was also higher in those who received immediate IMV, whilst patients who received NIV first reported a higher prevalence of non-COPD respiratory disease. These findings may be explained by clinicians’ reluctance to start IMV in patients with significant chronic lung disease, but as all patients have a diagnosis of CAP, they are of unclear significance. We report an NIV success rate of 59%, inclusive of patients who had NIV as a ceiling of therapy and were therefore not eligible for IMV, which is similar to previously reported results [11]. Overall, 45% of patients were successfully managed with NIV alone, and for these patients, the 28-day mortality and length of ICU stay were significantly better. Furthermore, unlike previous studies [18,20], we also found that there was no increased mortality when IMV was initiated following NIV failure. 

Our data suggest that the SOFA score may predict NIV failure in patients with severe CAP. The use of the SOFA score for this purpose has previously been described [14], although this study included patients with non-pulmonary sepsis. Nevertheless, the authors found that the SOFA score had an OR for NIV failure of 1.24, which is consistent with our finding of 1.33 (Table 4). Furthermore, the SOFA score upon ICU admission has an AUC of 0.75 in our ROC analysis, and this improves to 0.82 after 24 h. Overall, a SOFA score ≤ 4 upon ICU admission has a sensitivity and specificity for predicting NIV success of 61% and 72%, respectively. These results suggest that most patients who benefit from NIV may be identified early using the SOFA score upon admission. 

The HACOR score was developed to predict NIV failure in hypoxaemic ARF [22]. In patients with pneumonia, the HACOR score has been variously reported to have an AUC for NIV failure of 0.88 to 0.93 after 1 h of NIV [22,23]. However, we found that the HACOR score had an AUC of 0.66 for NIV failure upon ICU admission, which improved to 0.71 after 2 h of NIV (Table 4). In our analysis, a HACOR score ≤ 5 after 2 h of NIV had a sensitivity and specificity of 53% and 85%, respectively. We also evaluated the predictive ability of the recently updated HACOR score [24], which incorporates the SOFA score as well as binary variables, including the presence of immunosuppression, septic shock, acute respiratory distress syndrome (ARDS) or cardiogenic pulmonary oedema (CPE). We found that the updated HACOR score had a better AUC for NIV failure of 0.72 upon ICU admission and 0.76 after 2 h. However, a major disadvantage of the updated HACOR score is that a chest radiograph is required, the interpretation of which introduces bias and limits the ability to perform serial measurements.

Our results have several limitations. As a single-center retrospective study, our sample size was limited, and this, in conjunction with significant collinearity between variables, meant we chose not to perform multivariate analysis. Furthermore, all treatment decisions were made by the clinical team, and our analysis is unlikely to account for all factors considered by them at the time. For example, it is possible that some patients were started on NIV as a bridging therapy whilst preparations for IMV were ongoing. We have also been unable to report any data on NIV tidal volume, which has previously been associated with NIV failure [34]. Our results should also be interpreted in the context of how ICU care is utilized in the United Kingdom. Our practice includes discussions with patients regarding appropriate levels of therapy and ceilings of care that incorporate their wishes, pre-morbid functional status and frailty and the ability to recover from critical illness following invasive mechanical ventilation. In addition, HACOR data after 2 h of NIV were only available for approximately 80% of patients. This was largely because arterial blood gases were not repeated, which we hypothesize is more likely to have occurred in patients who were improving clinically. We were also unable to perform a multivariate analysis due to the small sample size. Nevertheless, our study suggests that NIV can be used as an initial respiratory support intervention for hypoxemic patients with severe community-acquired pneumonia. While NIV failure outcomes were comparable to those of IMV, NIV success had significantly better ICU outcomes. This suggests that using NIV to optimize oxygenation in severe pneumonia may be beneficial. However, careful patient selection is required, with consideration to ensure that the NIV is only administered in appropriate areas where at-risk patients can be offered immediate access to IMV without undue delay.

## 5. Conclusions

NIV may be used as initial respiratory support for patients with community-acquired pneumonia and hypoxemic respiratory failure. The NIV failure rate in this setting was 40.6%. Successful NIV was associated with much better outcomes when compared to immediate mechanical ventilation or patients who had NIV failure. Our results suggest that SOFA and HACOR scores can be used to identify patients who are likely to benefit from NIV early in their ICU admission. A SOFA score ≤ 4 upon ICU admission and a HACOR score ≤ 5 after 2 h of NIV are both predictive of NIV success. 

## Figures and Tables

**Table 1:** Patient characteristics, laboratory tests, prognostic scores and outcomes according to 28-day mortality.

Variable	All Patients (n = 140)	Survivors (n = 105)	Non-Survivors (n = 35)	*p*
Age, years (IQR)	65 (54–73)	64 (49–72)	69 (61–80)	0.0024 *
Charlson comorbidity index	4 (2–5)	3 (2–4)	4 (3–5)	0.0015 *
Sex				
Male, n (%)	76 (54)	55 (52)	21 (60)	0.5571
Female, n (%)	64 (46)	50 (48)	14 (40)	0.5571
Comorbidities				
IHD, n (%)	22 (16)	13 (12)	9 (26)	0.1042
CCF, n (%)	9 (6)	6 (6)	3 (9)	0.6907
COPD, n (%)	33 (24)	24 (23)	9 (26)	0.8186
Other respiratory, n (%)	31 (22)	25 (24)	6 (17)	0.4872
Diabetes mellitus, n (%)	33 (24)	27 (26)	6 (17)	0.3634
Cerebrovascular, n (%)	13 (9)	9 (9)	4 (11)	0.7369
CKD, n (%)	9 (6)	5 (5)	4 (11)	0.2274
Active cancer, n (%)	21 (15)	13 (12)	8 (23)	0.1708
Immunocompromise, n (%)	27 (19)	18 (17)	9 (26)	0.3227
** de novo ARF, n (%)	82 (59)	60 (57)	22 (63)	0.6923
Laboratory Tests				
PaO_2_/FiO_2_ ratio (mmHg)	151 (111–203)	153 (112–199)	146 (87–214)	0.6455
PaCO_2_ (mmHg)	40 (33–56)	39 (34–55)	40 (31–52)	0.4413
WCC (10^9^/L)	13.7 (8.9–21.4)	12.4 (8.5–18.9)	17.9 (10.4–23.7)	0.0332 *
Neut/Lymph ratio	11.4 (6.7–22.4)	11.1 (6.6–19.6)	16.4 (8.1–33.4)	0.1389
CRP (mg/L)	150 (48–259)	135 (40–234)	187 (114–266)	0.1615
Urea (mmol/L)	8.0 (5.1–12.1)	7.8 (5.1–12.0)	8.9 (5.1–12.7)	0.6672
Creatinine (μmol/L)	82 (64–122)	80 (64–120)	82 (59–123)	0.9203
Bilirubin (μmol/L)	12 (7–20)	12 (7–18)	12 (9–23)	0.3371
INR	1.3 (1.1–1.6)	1.3 (1.1–1.5)	1.4 (1.2–1.8)	0.2801
Laboratory Proven Infections				
Bacterial only, n (%)	34 (24)	22 (21)	12 (34)	0.1178
Viral only, n (%)	27 (19)	22 (21)	5 (14)	0.4656
Mixed, n (%)	10 (7)	9 (9)	1 (3)	0.4511
Prognostic Scores				
APACHE II score	19 (15–23)	18 (14–22)	23 (18–25)	0.0011 *
SOFA score	6 (3–10)	6 (3–10)	7 (5–11)	0.0930

Abbreviations: Ischemic heart disease (IHD), congestive cardiac failure (CCF), chronic obstructive pulmonary disease (COPD), white cell count (WCC), C Reactive Protein (CRP), International Normalized Ratio (INR), interquartile range (IQR), Acute Physiology and Chronic Health Evaluation II (APACHE II), Sequential Organ Failure Assessment (SOFA). Footnotes: data presented as number (%) or median (IQR). * *p* < 0.05. ** no prior chronic respiratory or cardiac disease.

**Table 2:** Patient characteristics, laboratory tests, prognostic scores and outcomes according to first respiratory support.

Variable	IMV 1st (n = 34)	NIV 1st (n = 106)	*p*
Age, years (IQR)	71 (63–78)	63 (51–72)	0.0232 *
Charlson comorbidity index	4 (2–4)	3 (2–5)	0.4593
Sex			
Male, n (%)	20 (59)	56 (53)	0.5602
Female, n (%)	14 (41)	50 (47)	0.5602
Comorbidities			
IHD, n (%)	7 (21)	15 (14)	0.4185
CCF, n (%)	2 (6)	7 (7)	1.000
COPD, n (%)	7 (21)	26 (25)	0.8169
Other respiratory, n (%)	3 (9)	28 (26)	0.0337 *
Diabetes, n (%)	6 (18)	27 (25)	0.4866
Cerebrovascular, n (%)	5 (15)	8 (8)	0.3048
CKD, n (%)	1 (3)	8 (8)	0.6878
Active cancer, n (%)	2 (6)	19 (18)	0.1032
Immunocompromise, n (%)	3 (9)	24 (23)	0.0852
de novo ARF, n (%)	26 (76)	56 (53)	0.0168 *
Laboratory Tests			
PaO_2_/FiO_2_ ratio (mmHg)	134 (86–183)	155 (112–206)	0.1285
PaCO_2_ (mmHg)	46 (39–59)	38 (33–52)	0.0385 *
pH	7.261 (7.186–7.368)	7.400 (7.300-7.447)	<0.0001 *
WCC (10^9^/L)	20.2 (9.2–22.8)	12.3 (8.8–18.5)	0.0251 *
Neut/Lymph ratio	15.9 (9.0–25)	11.1 (6.6–22.1)	0.1615
CRP (mg/L)	137 (28–253)	153 (55–257)	0.7188
Urea (mmol/L)	9.1 (5.2–14.5)	7.7 (5.2–12.0)	0.1471
Creatinine (μmol/L)	116 (69–163)	79 (63–104)	0.0561
Bilirubin (μmol/L)	14 (10–22)	12 (7–20)	0.1707
INR	1.3 (1.1–1.6)	1.3 (1.1–1.6)	0.5823
Prognostic Scores			
APACHE II score	20 (15–24)	19 (14–23)	0.1310
SOFA score	11 (10–14)	5 (3–7)	<0.0001 *
Outcomes			
ICU mortality, n (%)	10 (29)	14 (13)	0.0378 *
28-day mortality, n (%)	12 (35)	23 (22)	0.1178
Required CV support, n (%)	28 (82)	43 (41)	<0.0001 *
Required RRT, n (%)	9 (26)	11 (10)	0.0264 *
ICU days, days (IQR)	8 (5–18)	5 (3–9)	0.0056 *
Hospital days, (days (IQR)	20 (10–42)	14 (8–26)	0.1615

Abbreviations: ischemic heart disease (IHD), congestive cardiac failure (CCF), chronic obstructive pulmonary disease (COPD), white cell count (WCC), C Reactive Protein (CRP), International Normalized Ratio (INR), Acute Physiology and Chronic Health Evaluation II (APACHE II), Sequential Organ Failure Assessment (SOFA). Footnotes: data presented as number (%) or median (IQR). * *p* < 0.05.

**Table 3:** Prognostic variables at longitudinal timepoints and outcomes according to NIV success or failure.

Variable	NIV Success (n = 63)	NIV Failure (n = 43)	*p*
Upon ICU Admission			
pH	7.406 (7.335–7.450)	7.362 (7.259–7.433)	0.0767
PaO_2_/FiO_2_ ratio (mmHg)	155 (114–213)	155 (111–196)	0.3472
PaCO_2_ (mmHg)	38 (32–51)	40 (32–58)	0.2585
SOFA score	4 (3–6)	7 (4–10)	<0.0001 *
HACOR	5 (3–6)	6 (5–13)	0.0067 *
uHACOR	9.0 (6.5–12.5)	13.0 (10.0–20.0)	<0.0001
ROX Index	7.58 (5.42–10.83)	5.99 (4.36–9.48)	0.1936
After 2 h			
pH	7.390 (7.304–7.439)	7.326 (7.251–7.448)	0.3898
PaO_2_/FiO_2_ ratio (mmHg)	156 (116–233)	125 (90–178)	0.0615
PaCO_2_ (mmHg)	40 (35–53)	40 (31–49)	0.5287
HACOR score	5 (3–10)	10 (6–16)	0.0012 *
uHACOR	11.0 (7.0–14.5)	17.0 (12.5–25.0)	<0.0001 *
ROX Index	8.15 (5.60–12.09)	6.52 (4.32–9.31)	0.0549
After 24 h			
SOFA score	3 (2–5)	8 (6–11)	<0.0001 *
Outcomes			
ICU mortality, n (%)	0 (0)	14 (33)	<0.0001 *
28-day mortality, n (%)	8 (13)	15 (35)	0.0085 *
Required CV support, n (%)	9 (14)	34 (79)	<0.0001 *
Required RRT, n (%)	3 (5)	8 (19)	0.0471 *
ICU days, days (IQR)	4 (3–7)	8 (4–16)	0.0002 *
Hospital days, days (IQR)	13 (8–21)	18 (10–36)	0.0989

Abbreviations: Acute Physiology and Chronic Health Evaluation II (APACHE II), Sequential Organ Failure Assessment (SOFA), Heart Rate, Acidosis, Consciousness, Oxygenation and Respiratory Rate (HACOR), cardiovascular (CV), renal replacement therapy (RRT). Footnotes: data presented as number (%) or median (IQR). * *p* < 0.05.

**Table 4:** Prognostic variables with associated odds ratios and AUCs for NIV failure.

Prognostic Score	OR (95% CI, *p*)	AUC (95% CI, *p*)	Maximum Youden’s Index	Optimum Threshold
Upon ICU Admission				
HACOR	1.14 (1.04–1.24, 0.004 *)	0.655 (0.545–0.765, 0.006)	0.250	6.5
uHACOR	1.14 (1.06–1.23, <0.001 *)	0.717 (0.617–0.818, <0.001)	0.354	9.25
ROX Index	0.94 (0.86–1.04, 0.237)	0.589 (0.458–0.721, 0.184)	0.197	5.28
SOFA	1.33 (1.15–1.54, <0.001 *)	0.749 (0.655–0.844, <0.001)	0.372	5.50
APACHE II	1.08 (1.00–1.16, 0.042 *)	0.639 (0.520–0.758, 0.022)	0.319	20.5
After 2 h				
HACOR	1.13 (1.04–1.22, 0.004 *)	0.711 (0.601–0.821, <0.001)	0.384	5.50
uHACOR	1.15 (1.07–1.24, <0.001 *)	0.762 (0.660–0.863, <0.001)	0.390	12.25
ROX Index	0.92 (0.85–1.00, 0.050 *)	0.619 (0.502–0.736, 0.047)	0.218	8.20
After 24 h				
SOFA	1.52 (1.27–1.81, <0.001 *)	0.819 (0.725–0.914, <0.001)	0.592	5.50
Change–ICU admission to 2 h				
HACOR	1.02 (0.92–1.34, 0.651)	0.544 (0.417–0.670, 0.497)	0.162	1.50
uHACOR	1.03 (0.93–1.23, 0.598)	0.540 (0.411–0.668, 0.546)	0.196	1.50
ROX Index	0.92 (0.85–0.99, 0.045 *)	0.633 (0.515–0.751, 0.027)	0.261	0.69
Change–ICU admission to 24 h				
SOFA	1.29 (1.07–1.55, 0.007 *)	0.700 (0.587–0.813, 0.001)	0.311	1.50

Abbreviations: odds ratio (OR), area under receiver operating characteristic curve (AUC), Heart Rate, Acidosis, Consciousness, Oxygenation and Respiratory Rate (HACOR) score, updated HACOR score (uHACOR), Ratio of Oxygen Saturation (ROX) index, Sequential Organ Failure Assessment (SOFA), Acute Physiology and Chronic Health Evaluation II (APACHE II). * *p* < 0.05.

## Data Availability

Upon request.
